# Targeting accuracy of neuronavigation: a comparative evaluation of an innovative wearable AR platform vs. traditional EM navigation

**DOI:** 10.3389/fdgth.2024.1500677

**Published:** 2025-01-14

**Authors:** Marina Carbone, Nicola Montemurro, Nadia Cattari, Martina Autelitano, Fabrizio Cutolo, Vincenzo Ferrari, Emanuele Cigna, Sara Condino

**Affiliations:** ^1^ Department of Information Engineering, University of Pisa, Pisa, Italy; ^2^ EndoCAS Interdipartimental Center, University of Pisa, Pisa, Italy; ^3^ Department of Neurosurgery, Azienda Ospedaliero Universitaria Pisana, Pisa, Italy; ^4^ Department of Translational Research and New Technologies in Medicine and Surgery, University of Pisa, Pisa, Italy

**Keywords:** neuronavigation, augmented reality navigation, targeting accuracy, head mounted displays, neurosurgery, surgical navigation

## Abstract

Wearable augmented reality in neurosurgery offers significant advantages by enabling the visualization of navigation information directly on the patient, seamlessly integrating virtual data with the real surgical field. This ergonomic approach can facilitate a more intuitive understanding of spatial relationships and guidance cues, potentially reducing cognitive load and enhancing the accuracy of surgical gestures by aligning critical information with the actual anatomy in real-time. This study evaluates the benefits of a novel AR platform, VOSTARS, by comparing its targeting accuracy to that of the gold-standard electromagnetic (EM) navigation system, Medtronic StealthStation^®^ S7^®^. Both systems were evaluated in phantom and human studies. In the phantom study, participants targeted 13 predefined landmarks using identical pointers to isolate system performance. In the human study, three facial landmarks were targeted in nine volunteers post-brain tumor surgery. The performance of the VOSTARS system was superior to that of the standard neuronavigator in both the phantom and human studies. In the phantom study, users achieved a median accuracy of 1.4 mm (IQR: 1.2 mm) with VOSTARS compared to 2.9 mm (IQR: 1.4 mm) with the standard neuronavigator. In the human study, the median targeting accuracy with VOSTARS was significantly better for selected landmarks in the outer eyebrow (3.7 mm vs. 6.6 mm, p = 0.05) and forehead (4.5 mm vs. 6.3 mm, p = 0.021). Although the difference for the pronasal point was not statistically significant (2.7 mm vs. 3.5 mm, p = 0.123), the trend towards improved accuracy with VOSTARS is clear. These findings suggest that the proposed AR technology has the potential to significantly improve surgical outcomes in neurosurgery.

## 1 Introduction

Neurosurgery is a complex and delicate field that requires high precision and accuracy. Surgeons now have access to various computer-assisted surgery (CAS) technologies that enable surgical planning based on preoperative imaging and, during surgery, provide intraoperative instrument guidance. Navigation technologies work by spatially linking the patient and surgical tools to the image data through “patient-to-image” registration. Neuronavigators have been widely used to guide brain biopsy (1), transphenoidal surgery for the resection of pituitary adenomas (2), and brain tumor resection (3).

According to the literature, neuronavigation offers several advantages to surgeons, such as precise planning of the incision and craniotomy, and the identification of small subcortical lesions (4). In addition to anatomical data, functional magnetic resonance imaging (5) and tractography information (6) are possible as overlays available during surgery.

Clinical demonstrations of the neuronavigation benefit include the study from Wirtz et al. (7) focusing on glioblastoma surgery. The study compared the impact of neuronavigation on time consumption, the extent of tumor removal, and survival. It showed that absolute and relative residual tumor volumes are significantly lower with neuronavigation, and patients operated with neuronavigation had longer survival (median 13.4 vs. 11.1 months). Additionally, neuronavigation increased the extent of tumor removal in glioblastoma resection without prolonging operating time.

Two main modalities of navigator tracking are currently adopted, namely optical (OTS) and electromagnetic tracking systems (EMTS). Both OTS and EMTS techniques have proven their value in neurosurgical navigators where they are equally popular. The main limitation of the OTS is the requirement for a direct line of sight between the camera, the patient reference frame (used for continuous tracking of anatomy during registration and navigation), and the probe during navigation. If there is a “line-of-sight occlusion,” the system cannot track and navigate. EMTS overcomes this limitation; indeed, no line of sight is needed between the transmitter (electromagnetic field generator) and the receiver (sensor coil integrated in the surgical tool). In addition, electromagnetic navigation probes have a more compact design, making them easier to use under a microscope. However, a disadvantage of EMTS is the need to position the electromagnetic field generator near the surgical target. Furthermore, the accuracy of EMTS systems can be affected by the proximity of ferromagnetic instruments; however, current evidence suggests that this issue rarely arises in clinical settings (3, 8).

In addition to the technical accuracy related to the tracking technologies employed, the accuracy of the neuronavigation can be affected by various sources of errors. Wang et al. (9) identified two groups of errors based on the neuronavigator working principle. Error Type I includes errors caused by differences between the anatomical structures in the images and the actual patient, such as brain deformation and low image resolution. Type II error involves errors in the transformation of the position of surgical tools from the patient space to the image space, including tracking errors and surgical toll calibration inaccuracy, as well as image-to-patient registration errors. In the past, neuronavigation relied on direct point matches of bone-implanted fiducials, which provided high accuracy but caused discomfort. Modern systems use paired point registration with adhesive markers and surface matching algorithms, which, although less accurate than registration based on implanted cranial markers, have proven to be suitable for daily use in most neurosurgical cases with a reported accuracy between 1.8 mm and 5 mm (10, 11).

Conventional navigators are also limited in terms of visualization ergonomics: they display the guidance information on an external screen, which means that surgeons often shift their attention between the surgical field and the monitor. Augmented reality (AR) visual interfaces provide a solution by presenting the surgeon with virtual information seamlessly blended with the patient’s anatomy (e.g., this can include displaying a planned cutting line to guide an osteotomy superimposed on the patient’s bony anatomy). This approach reduces cognitive load and improves information management in image-guided surgery. Head-mounted displays (HMDs) provide an ergonomic interface that maintains the user’s egocentric view of the surgical field. For this reason, they are considered the most ergonomic and effective medium for guiding procedures that are performed manually under the surgeon’s direct vision, such as procedures involving manual manipulation of exposed human tissue, including incisions in epithelial, muscle, or bone structures.

In recent years, burgeoning research interests have been devoted to developing AR-based neuronavigators (12–14), for example for surgical resection of intracranial meningiomas (15), providing insight into the disruptive potential of AR in neurosurgery. However, most studies on HMD are “proofs of concept” trials based on using a Microsoft HoloLens (16), a self-contained Optical See Through (OST) headset, outside its indication and despite the technological and human-factor limits that prevent achieving high accuracy levels. To list the most relevant: the perceptual conflicts between the view of the real world and the VR image (17), the small field of view (FoV), the sub-optimal ergonomics, and calibration issues to attain a robust VR-to-real alignment (18–20).

To the best of the authors’ knowledge, today, there is no neurosurgery-specific HMD designed to comply with medical device regulation that has been validated in a relevant environment for guiding high-precision tasks. To address this gap, we aim to test VOSTARS, a novel hybrid video and optical see-through HMD designed for precision surgery applications, in the field of neurosurgical oncology for targeting supratentorial tumors, both intraparenchymal and extra-axial tumors, including plaque cranial vault meningiomas. Developed within the Horizon 2020 project framework, VOSTARS has already demonstrated promise in guiding maxillofacial osteotomies (21). We further validated its navigation performance through recent in-vitro studies using patient-specific phantoms (22, 23). These studies yielded impressive results, with a mean real-to-virtual 3D target visualization error (TVE3D) of just 1.3 mm and a standard deviation of 0.6 mm. Additionally, user studies showed that subjects guided by VOSTARS could trace a remarkable 97% of a planned craniotomy trajectory within a 1.5 mm error margin, with an outstanding 92% achieving a 1 mm margin. These results were obtained using a skin-fixed dynamic reference frame (DRF) for real-time registration.

In this study, we aim to evaluate the targeting accuracy achievable with the VOSTARS system in combination with the aforementioned DRF, and compare its performance to that of a traditional commercial navigation system. The most widely used neuronavigation systems on the market are produced by leading companies such as Stryker, Brainlab, and Medtronic, with the latter two being particularly notable for their strong clinical presence (24). A literature review suggests that the navigational accuracy of Medtronic’s StealthStation, BrainLab’s VectorVision, and Stryker’s iNtellect systems is generally comparable (25). For this study, we have selected the Medtronic StealthStation^®^ S7^®^ as the benchmark, as it is available in the neurosurgery unit participating in the trial.

Tests were performed first on a patient-specific phantom, then we proceeded with a noninvasive study on nine volunteers. Trials are focused on the targeting of superficial landmarks because the ultimate goal is to demonstrate the applicability of the VOSTARS system in guiding complex craniotomy procedures. Recent literature studies suggest that planning and executing an appropriately positioned and sized craniotomy, is the central role of a neuronavigational system in neurosurgery (3).

## 2 Material and methods

### 2.1 Navigation systems

**Figure 1:**
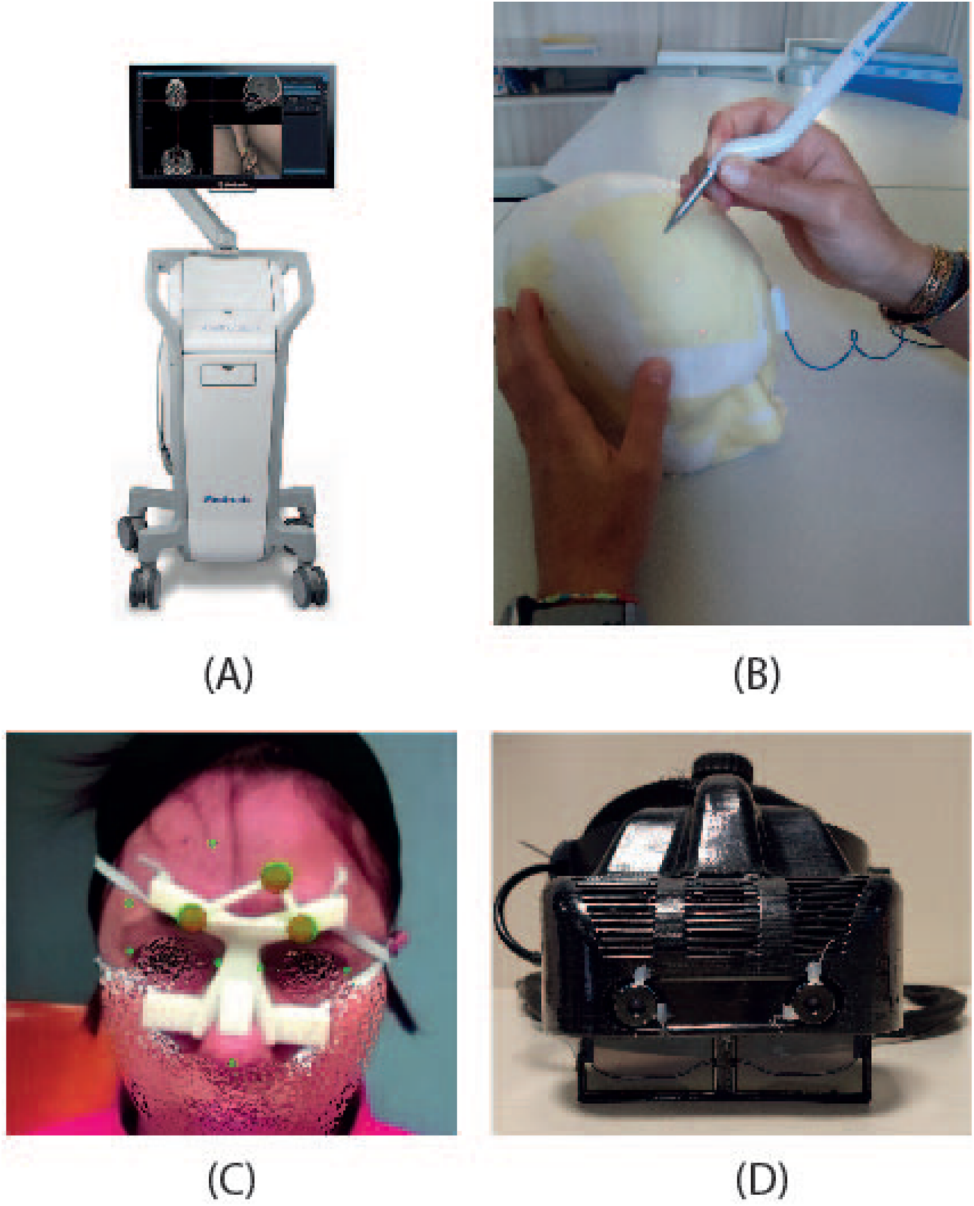
The figure depicts the two systems compared: **(A)** StealthStation S7 navigation system, and **(B)** a user performing the targeting experiment with the electromagnetic navigator. **(C)** the augmented reality scene visualized through VOSTARS, and **(D)** the VOSTARS visor.

The StealthStation^®^ S7^®^ System (Medtronic Inc., Louisville, CO, USA) is a popular commercial navigator (Figure 1A) that provides real-time surgical guidance by combining radiological images of the patient with real-time surgical tool tracking using optical or electromagnetic technology. The application software allows loading patient-specific CT or MR images acquired before surgery, or fluoroscopic images captured during surgery and displays them on the screen from various perspectives (such as axial, sagittal, coronal, and oblique). The surgeon can plan and save one or more surgical trajectories in the preoperative phase. Additionally, the surgeon can create and manipulate one or more 3D anatomical models to aid visualization. During surgery, the system continuously updates the position of the instruments on these radiological images by tracking the position of specialized surgical instruments in or on the patient’s anatomy using optical or electromagnetic tracking.

In 2013 (26), the technical accuracy of the StealthStation^®^ S7^®^ was assessed in a hospital setting using an ad-hoc designed phantom containing 51 target points. The OTS and EMTS measurement performances were estimated in a volume of 120 mm × 120 mm × 100 mm, roughly mimicking the size of the human head. The accuracy is calculated by evaluating the error in the distance of each target point to a reference point, both acquired with a navigated tooltip. The protocol does not require performing a registration procedure and repurges measured errors from human performance related to interface ergonomics and intrinsic user accuracy. Results showed that the technical accuracies of OTS and EMTS over the pre-determined volume are nearly equal: 0.20 mm ± 0.10 mm and 0.30 mm ± 0.13 mm, respectively.

Depending on the assessed device and methods, other studies evaluating the OTS and the EMTS technical accuracies have reported values up to 1.4 mm (26).

In our work, we utilized the EMTS functions of the StealthStation^®^ S7^®^ System. Indeed, this modality does not require rigid immobilization of the surgical area (e.g., using skull clamps) making it suitable for non-invasive testing on human volunteers in a non-surgical environment. This is allowed by the dynamic referencing function of the navigator: a proprietary patient reference frame (Figure 1C) is attached to the patient for the real-time update of the image-to-patient registration, thus ensuring accurate navigation.

The VOSTARS AR headset (Figure 1D) was created by modifying a commercial OST visor (ARS.30^®^ by Trivisio). This headset can provide optical and video see-through augmentations using two liquid-crystal (LC) optical shutters (FOS model by LC-Tec^®^) placed on top of the semi-transparent optical combiners of the visor. By adjusting the drive voltage, users can switch between a regular optical see-through (OST) view (with shutters open) and a video see-through (VST) camera-assisted view (with shutters closed). The ARS.30^®^ visor is equipped with dual SXGA OLED panels with 1280×1024 resolution, a 30° diagonal field of view, and a 3 cm eye relief. The OST display has an average angular resolution of approximately 1.11 arcmin/pixel, which is comparable to human visual acuity. The visor’s collimation optics were redesigned to have a focal length of about 40 cm. Additionally, the two optical engines of the visor are slightly toed-in, meaning that the optical axes of the two displays converge at approximately the focal length of the collimation optics. These features are crucial for reducing issues like vergence-accommodation conflict and focus rivalry (17) when the headset is used in the peripersonal space.

The visor is made up of a 3D-printed plastic frame that incorporates two LC shutters and houses a pair of world-facing RGB cameras (two USB 3.0 LI-OV4689 cameras by Leopard Imaging, both equipped with 1/3” OmniVision CMOS 4M pixels sensor (pixel size: 2μm) and an M12 lens with 6 mm focal length). These cameras are used for inside-out tracking and to provide the VST view. The cameras have a horizontal field of view of approximately $46^{\circ }$, corresponding to an average angular resolution of about 2.2 arcmin/pixel. The stereo camera pair is mounted on the top of the visor with an anthropometric interaxial distance of about 63μm to minimize the effect of camera-to-eye parallax. This setup achieves a quasi-orthostereoscopic perception of the scene under VST view.

**Figure 2:**
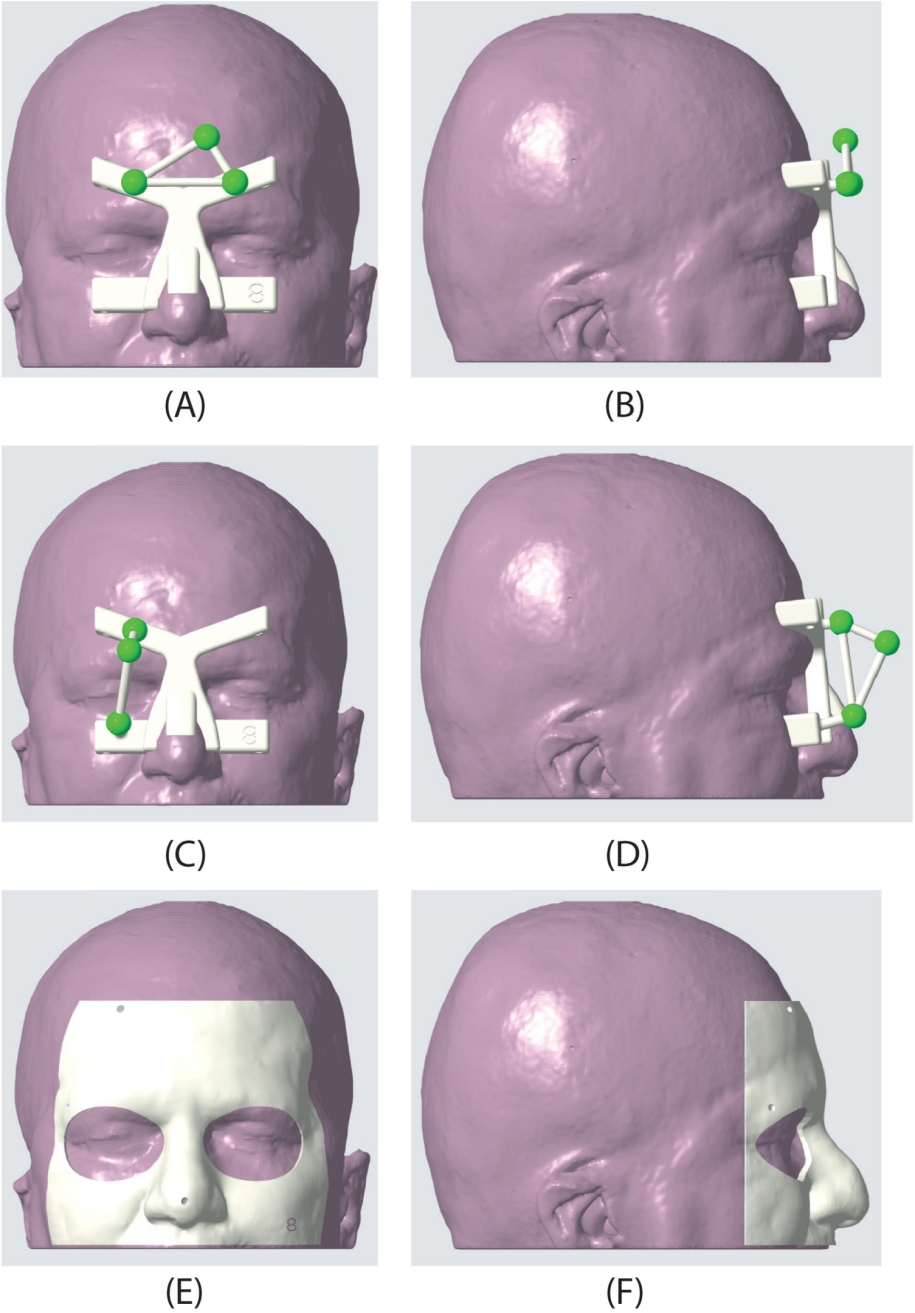
Dynamic reference frame (DRF) and patient-specific masks used in the AR experiment. The second row shows the frontal DRF used for the targets in the frontal, orbital, and nasal regions. **(A)** frontal view, **(B)** sagittal view. In the second row, the lateral DRF is shown; this DRF was used to target the points in the temporal region. **(C)** frontal view, **(D)** sagittal view. Finally, the third row presents one of the patient-specific masks used to evaluate the accuracy of landmark targeting. **(E)** frontal view, **(F)** sagittal view.

For neurosurgical applications, we designed a custom-made DRF (Figures 2A–D) to facilitate registration during procedures. This frame utilizes colored fiducial markers embedded within a 3D-printed patient-specific template. The biocompatible and sterilizable material used for printing, like MED610 from Stratasys, ensures patient safety. Pre-operative MRI scans guide the creation of this template, ensuring a customized fit for each patient’s face. Three strategically placed spherical markers, each 12 mm in diameter, serve as tracking markers during the registration process. The template’s design offers an intuitive registration experience due to its snug fit and the clear positioning of the markers on the face. The template’s shape and placement are designed based on facial anatomical landmarks that experience minimal deformation due to the thin underlying soft tissue layer, as detailed in (22).

### 2.2 Patient-specific phantom

**Figure 3:**
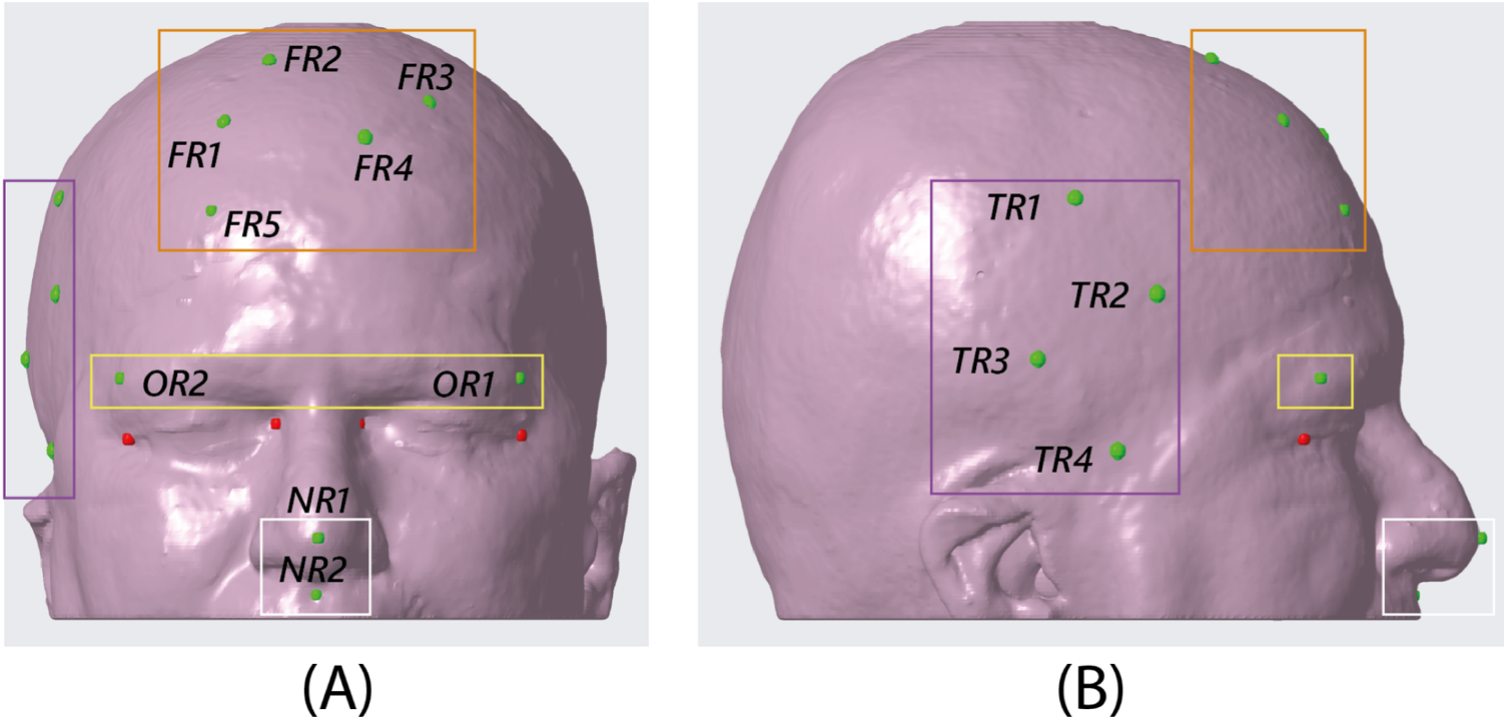
CAD view of the 13 target points identified on the phantom surface. The orange box encloses the five targets in the frontal region, the purple box the four targets in the temporal region, the yellow box the two targets in the orbital region, and the white box the two targets in the nasal region. NB. the red targets shown in the figure are the spheres designed at the canthi of the eyes as a sanity check for the proper placement of the dynamic reference frame in the AR experiment. **(A)** frontal view, **(B)** sagittal view.

In vitro experiments were performed on a patient-specific 3D-printed mannequin (Figure 1B). The 3D model of the mannequin was generated from a real MRI dataset (an axial spoiled gradient recalled acquisition in the steady-state (SPGR) sequence with a 0.5 mm × 0.5 mm × 0.6 mm resolution), segmented with a semi-automatic pipeline (27) to extract the head surface. 13 holes (5 points in the frontal region, 4 in the temporal region, 2 in the orbital region, and 2 in the nasal region) 1 mm in diameter were designed on the phantom surface to be used as targets for accuracy evaluation (Figure 3). Two DRFs were designed for the phantom and used during the navigation trials for aiding the VOSTARS navigation (Figures 2A–D). A 3D printer (Dimension Elite^®^) was used to turn the virtual model into a tangible replica made of acrylonitrile butadiene styrene.

**Figure 4:**
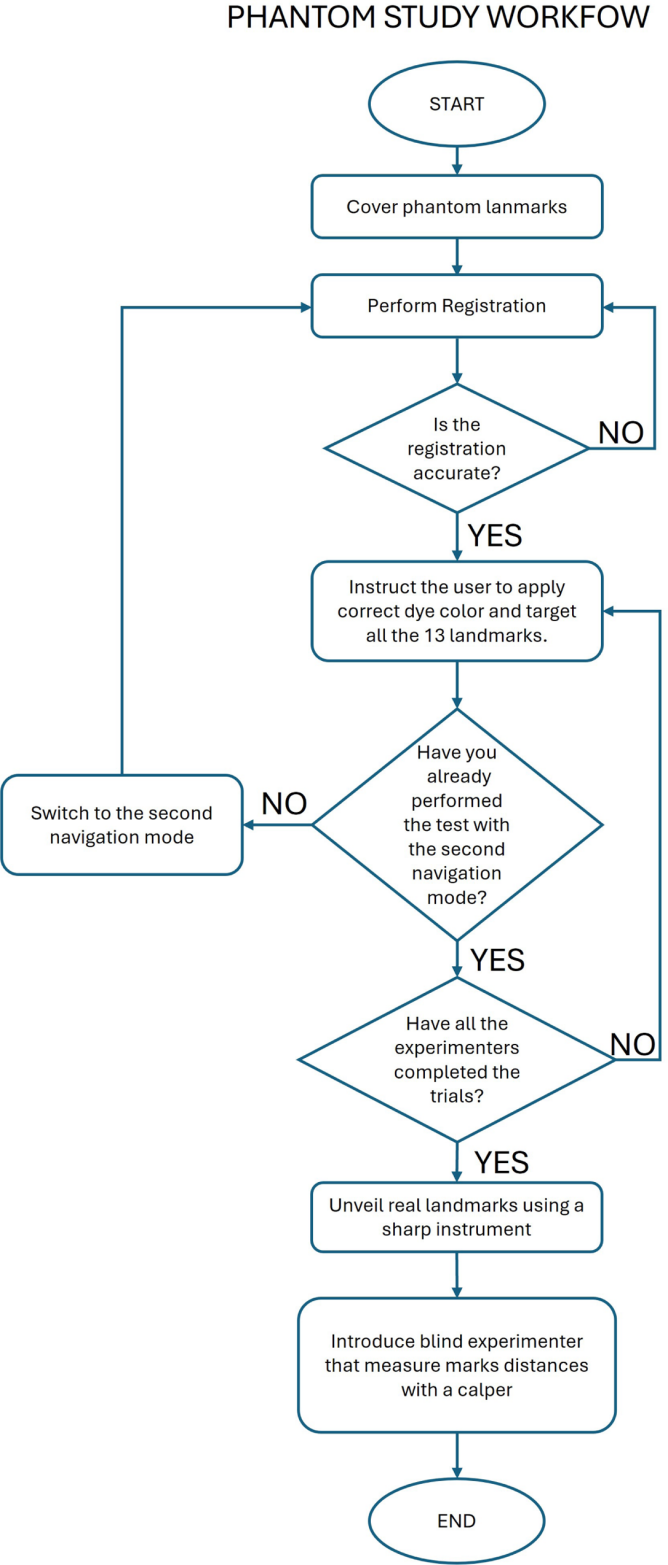
Graphical representation of the phantom trial workflow.

### 2.3 Volunteers for human study

**Figure 5:**
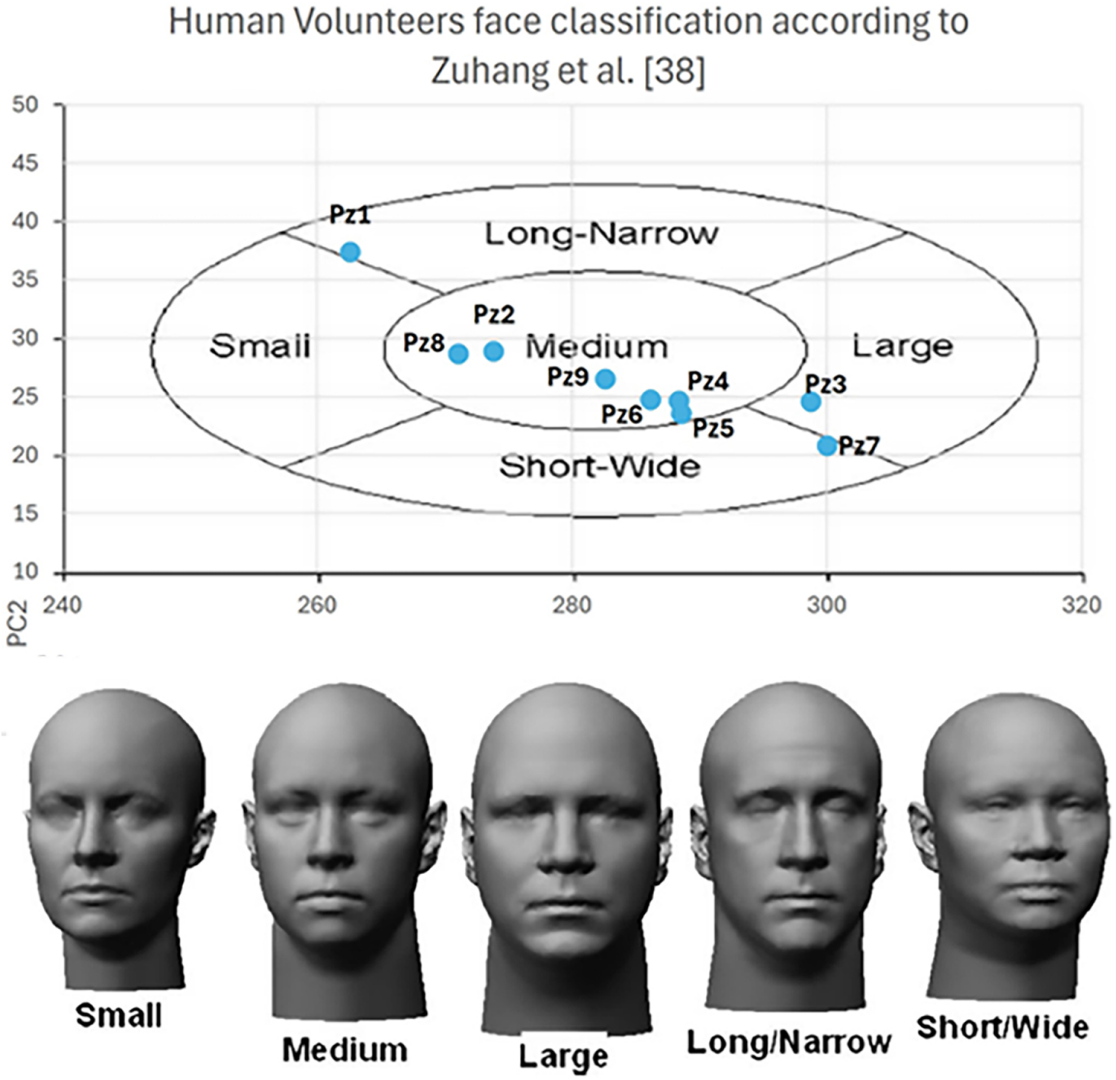
Human volunteers anthropometric data distribution among the face classification according to (28).

Nine volunteers were recruited among patients who underwent surgery through a standard craniotomy for brain tumor (grade I meningioma according to W.H.O. classification) exeresis at the Pisana University Hospital (Pisa, Italy). Inclusion criteria comprised a recent (less than 6 months) postoperative MRI performed, with an axial spoiled gradient recalled acquisition in the steady-state (SPGR) sequence with a 0.5 mm × 0.5 mm × 0.6 mm resolution. All recruited subjects were self-sufficient adults, able to provide informed consent, with no signs of recurrence on MRI. Table 1 reports the data of recruited subjects. Recruited volunteers’ anthropometric data for head and face dimensions include 4 out of the 5 (80%) “face type” classified in (28) that cover all the anthropometric characteristics of human beings. The distribution is shown in Figure 5.

**Table 1:** Demographics of volunteers recruited for the human-study.

General info	Values
Gender (number of male; number of female; number of non-binary)	(0; 9; 0)
Age (min; max; mean; STD)	(39; 76; 58.7; 12.4)
Time in months since last MRI acquisition (min; max; mean; STD)	(2; 6; 4.8; 1.9)

### 2.4 Subjects recruited to test the navigators

Four subjects aged between 28 and 42 with normal visual acuity or corrected-to-normal visual acuity (with the aid of contact lenses) were recruited. Table 2 reports the participants’ demographics) to perform navigation trials with the VOSTARS system and the StealthStation^®^ S7. The subjects were four biomedical engineers skilled in surgical navigation systems development and testing. They all signed a dedicated informed consent containing general information about the commercial navigation system used and the VOSTARS AR platform as well as the aim of the trial.

**Table 2:** Demographics of users recruited for testing the navigation systems in the phantom-study.

General info	Values
Gender (male; female; non-binary)	(0; 4; 0)
Age (min; max; mean; STD)	(28; 42; 35.8; 6.8)
Visual Acuity (normal; corrected to normal)	(4; 1)
Experience with VOSTARS in-vitro (none; limited; familiar; experienced)	(0; 0; 1; 3)
Experience with StealthStation in-vitro (none; limited; familiar; experienced)	(0; 0; 2; 2)
Experience with EM navigation in-vitro (none; limited; familiar; experienced)	(0; 0; 2; 2)

### 2.5 Phantom experiment protocol

Subjects were randomly assigned to use the VOSTARS system or the StealthStation^®^ first and were instructed to target the 13 phantom landmarks (target holes) using the two navigators. In both cases, subjects were instructed to use the pointer of the StealthStation^®^ to avoid introducing distortions related to the dimensional characteristics of the targeting tool. The pointer features a spherical tip with a 1 mm size, matching the target holes.

The protocol for both navigation methods is reported below and shown in Figure 4.

**Figure 6:**
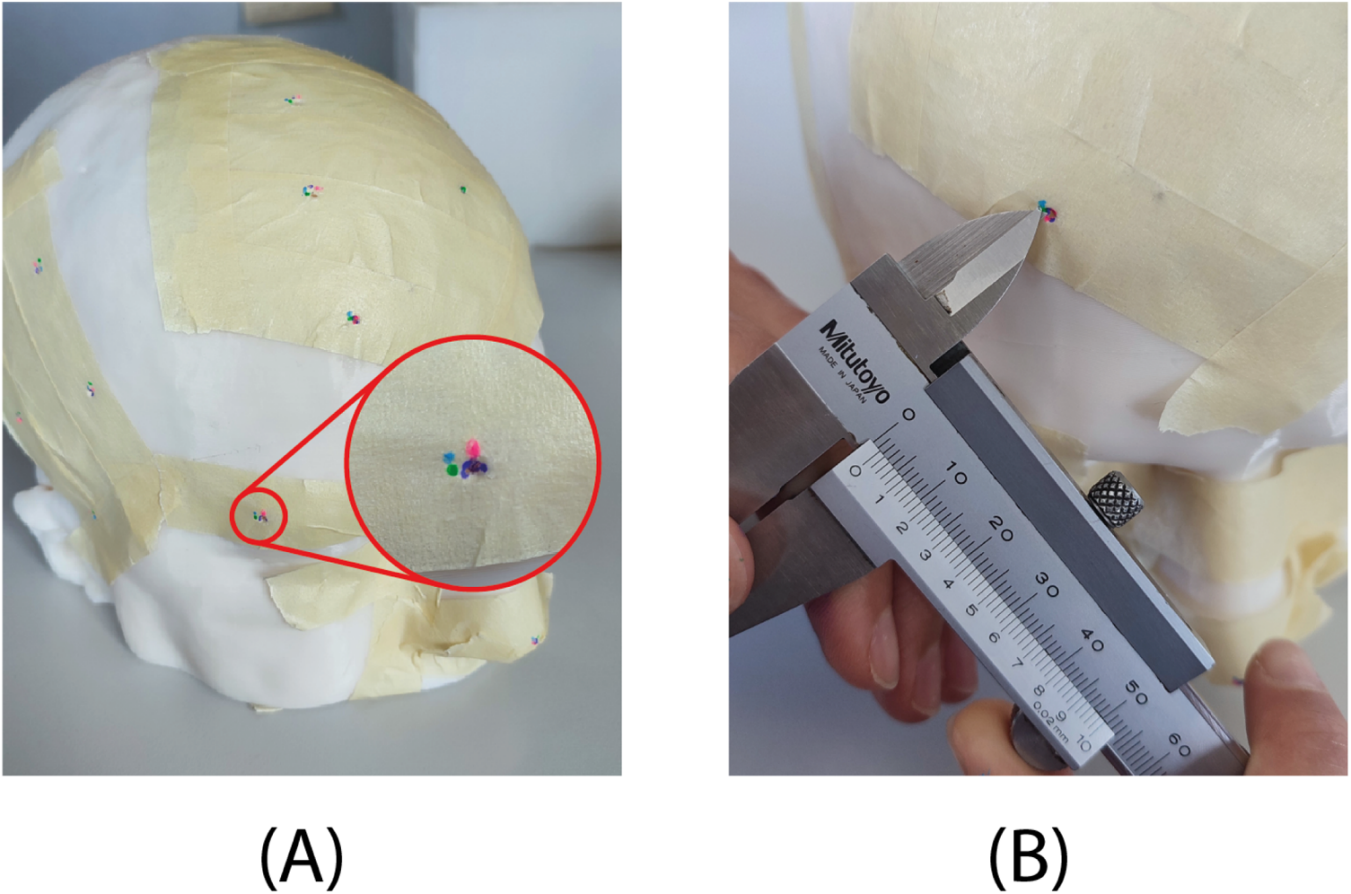
Results of the test on phantom. **(A)** Each of the four users indicated the target location with a different color. For the zoomed-in target, a user (purple) accurately marked the location at the hole embedded in the phantom, while the other three users (green, pink, and blue) were slightly off-target. **(B)** Example of error measurement with the caliper.

• Step 1: Cover the phantom landmarks with adhesive tape to hide their position (Figure 6A).
• Step 2: Perform registration.
• Step 3: Check the accuracy of the registration and repeat step 2 until the registration is successfully performed.
• Step 4: Instruct the user to dip the pointer tip into liquid dye, target each landmark as shown by the navigator, and make a colored mark at each landmark.
• Step 5: Repeat Step 4 for each subject.
• Step 6: Use a sharp instrument to uncover the actual position of each landmark.
• Step 7: An experimenter, blind to the navigation method, uses a caliper to measure the distance of each mark from the actual landmark. For marks completely inside the holes (not visible on the adhesive tape after Step 5), record an accuracy of 0.5 mm (Figure 6B).

During Step 2, in the trials using StealthStation^®^ navigation, the Medtronic patient reference frame was attached to the phantom’s forehead. Initial registration was achieved using surface-matching, which aligns a cloud of digitized points on the patient’s scalp with a volume rendering of the imaging data (29). This registration method is popular in neuronavigation due to its advantages over the more accurate but less convenient procedure based on point-pair matching with adhesive markers. These advantages include ease of use, not requiring additional imaging with markers in place and cost-effectiveness. In VOSTARS navigation trials, the DRF was positioned on the phantom’s face and held in place by elastic bands. The registration was obtained implicitly, as the DRF fits the phantom’s face, providing pose registration (22).

As for Step 3, the registration accuracy estimated by the StealthStation^®^ navigation software was utilized. A 1 mm error was selected as a threshold to consider the registration accurate enough for the trials. Additionally, a registration “sanity-check” procedure was performed (30): the experimenter “touched” some easily identifiable anatomical landmarks with the tracked probe, and the relative positioning of the probe and the anatomical model was checked on the navigator screen. The landmarks used were the canthi of the eyes and the pronasal point at the anterior apex of the nose. During VOSTARS trials, a sanity check procedure was employed to verify the proper placement of the DRF. AR spheres were designed at the canthi of the eyes, allowing the experimenter to visually estimate the template placement from different viewpoints. The positioning of the template was corrected until the AR spheres appeared perfectly aligned with the corresponding anatomical landmarks.

### 2.6 Human study protocol

The human study was focused on evaluating the accuracy of a single user in targeting 3 landmarks on the face of the 10 recruited volunteers. The pronasal point (PN), a point on the outer eyebrow (OE), and a point on the forehead (FH) were used as navigation targets (Figure 1C). Each recruited subject was provided with a custom mask with holes at the three target points to pinpoint their exact locations based on their selection on the MRI dataset. The custom mask, as the DRF, was designed starting from the segmentation of the MRI images to easily fit the patient’s face, in a unique and stable position. Compared with DFR, the masks are characterized by a larger fitting area. The starting navigation modality was randomly chosen for each patient, and the StealthStation^®^ pointer was used during both navigation trials.

**Figure 7:**
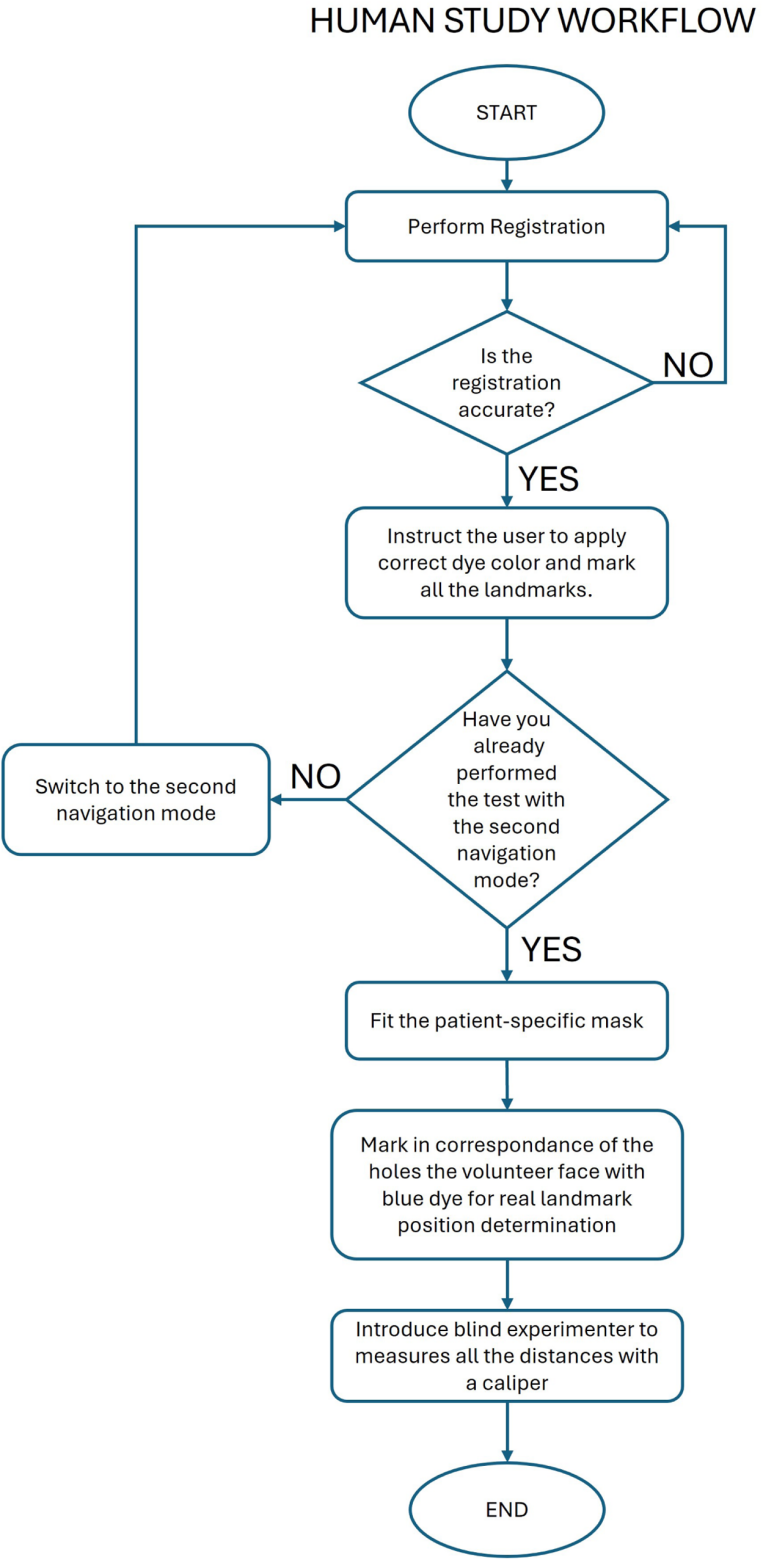
Graphical representation of the human trial workflow.

The protocol of the study is reported below and depicted in Figure 7.

• Step 1: Start with the first navigation modality and perform the registration procedure.
• Step 2: Check the accuracy of the registration and repeat step 1 until the registration is successfully performed.
• Step 3: Instruct the user to dip the pointer tip into liquid green dye, target each landmark as shown by the navigator, and make a mark at each landmark.
• Step 4: Switch to the second navigation mode and perform the registration procedure.
• Step 5: Check the accuracy of the registration and repeat step 4 until the registration is successfully performed.
• Step 6: Instruct the user to dip the pointer tip into red liquid dye, target each landmark as shown by the navigator, and make a mark at each landmark.
• Step 7: Fit the patient-specific mask on the volunteer’s face.
• Step 8: Use the mask holes as a guide to mark the volunteer’s face with blue, indicating the planned position of the three targets.
• Step 9: An experimenter, unaware of the randomization order, uses a caliper to measure the distance of each green and red mark from the corresponding blue mark.

Registration and sanity check procedures were the same as those used in the phantom tests.

### 2.7 Statistical analysis

The GNU PSPP 2.0.1 software was used to perform statistical analysis of data. The results of the targeting accuracy estimation were summarized in terms of median and interquartile range (IQR). A Wilcoxon signed-rank test was used to assess whether there was a significant difference in the users’ targeting accuracy based on the navigator used. A p-value less than or equal to 0.05

## 3 Results

### 3.1 Phantom study results

Table 3 shows the performance results of four subjects who tested the VOSTARS system and the StealthStation^®^ S7^®^ for targeting 13 different landmarks on the phantom. These landmarks include five points in the frontal region (FR), four in the temporal region (TR), two in the orbital region (OR), and two in the nasal region (NR) (Figure 3).

**Table 3:** Results of phantom-study: comparison of Vostars and StealthStation^®^ across various landmarks and users.

Landmark	Vostars						StealthStation^®^					
	User 1	User 2	User 3	User 4	Median	IQR	User 1	User 2	User 3	User 4	Median	IQR
FR 1	1.4	1.4	1.7	1.6	1.5	0.2	2.1	3.0	2.5	2.4	2.5	0.3
FR 2	1.4	1.8	2.7	1.9	1.9	0.4	3.1	3.1	3.1	3.0	3.1	0.0
FR 3	0.5	0.5	0.5	0.5	0.5	0.0	2.1	3.3	1.8	2.3	2.2	0.5
FR 4	0.5	0.5	0.5	0.5	0.5	0.0	2.0	2.0	3.1	2.5	2.3	0.7
FR 5	0.5	0.5	1.5	0.7	1.6	0.6	2.0	2.4	2.4	2.3	3.9	0.1
TR 1	0.5	2.5	1.5	1.6	1.6	0.6	3.9	4.2	3.9	3.8	3.8	0.8
TR 2	0.5	1.6	2.7	1.6	2.2	1.2	2.9	3.6	4.7	4.0	4.4	0.0
TR 3	1.9	1.7	4.5	2.5	2.2	0.8	4.4	4.4	4.4	4.2	3.0	0.1
TR 4	1.2	2.0	3.6	2.3	1.1	0.3	2.9	3.0	3.0	2.8	2.2	0.3
NR 1	0.5	1.1	1.5	1.0	0.8	0.7	1.4	2.2	2.3	2.1	1.6	0.2
NR 2	0.5	0.5	1.6	1.0	1.0	1.4	1.7	1.7	1.4	1.5	2.0	0.3
OR 1	0.5	0.5	3.3	1.4	1.5	0.7	1.7	2.1	2.1	1.9	3.6	0.3
OR 2	0.5	1.7	2.1	1.3	0.6	0.4	3.2	3.5	4.1	3.6	2.4	0.2

Overall, users achieved 1.4 mm median accuracy (IQR:1.2 mm) with VOSTARS and 2.9 mm (IQR:1.4 mm) with the standard neuronavigator. The results of the Wilcoxon signed-rank test indicate a statistically significant difference in the performance of all users according to the navigation modality (User1 p = 0.001, User2 p = 0.001, User3 p = 0.033, User4 p = 0.001).

### 3.2 Human study results

Table 4 shows the performance results of a single subject involved in testing the VOSTARS system and the StealthStation^®^ S7^®^ for targeting three facial landmarks (pronasal point (PN), a point on the outer eyebrow (OE), and a point on the forehead (FH)) in nine volunteers.

**Table 4:** Results of human-study: comparison of Vostars and StealthStation^®^ for various facial points.

Subject #	Pronasal point		Outer eyebrow		Forehead	
	Vostars	StealthStation^®^	Vostars	StealthStation^®^	Vostars	StealthStation^®^
1	5.8	10.1	6.2	6.7	9.1	10.8
2	3.0	3.5	4.8	16.6	4.5	5.8
3	1.6	1.3	2.6	1.4	3.3	0.3
4	4.8	4.0	2.5	4.4	2.8	4.4
5	0.7	2.4	2.3	5.7	1.2	1.7
6	5.8	10.1	2.7	6.6	8.7	10.4
7	2.7	2.4	3.8	7.6	2.0	6.3
8	2.0	4.4	3.7	4.0	7.0	10.6
9	2.0	2.0	4.0	8.5	8.8	15.2
Median	2.7	3.5	3.7	6.6	4.5	6.3
IQR	2.8	2.0	1.4	3.2	5.9	6.2

The median targeting accuracy using the VOSTARS system was better than that obtained with the StealthStation^®^ for all three facial landmarks (2.7 mm vs. 3.5 for PN; 3.7 mm vs. 6.6 for OE; 4.5 mm vs. 6.3 for OE). The Wilcoxon signed-rank test results revealed a statistically significant difference in targeting accuracy only for the OE and FH landmarks (PN p = 0.123; OE p = 0.05; FH p = 0.021).

## 4 Discussion

This work focuses on a comparative evaluation of an innovative HMD AR navigation system vs. traditional EM navigation for neurosurgery.

Literature studies on AR-HMD for neuronavigation mainly concentrate on the use of the Microsoft HoloLens, a general-purpose headset, not specifically designed for surgery. A recent clinical trial in neuro-oncology with this device showed that it can enable the surgeon to understand the relationship of the pathology with the surrounding structures (31). Table 5 summarizes the accuracy results of previous literature studies on wearable AR neuronavigation, focalized on cranial procedures. The experimental setups and error metrics reported in the different studies vary significantly, making direct comparisons with our results challenging. Nevertheless, it is worth highlighting that in prior studies involving the Microsoft HoloLens, the average display error consistently exceeded 4 mm for both fiducial markers (Fiducial Registration Error, FRE) and targets (Target Registration Error, TVE). Additionally, the TDE, which is further influenced by the user’s skill level, has similarly not demonstrated lower values in those studies.

**Table 5:** FRE, Fiducial registration error as root mean square distance between real fiducial positions and the associated virtual homologous; TVE2D, Target visualization error estimated as reprojection error (commonly in px) in 2D onto the displayed AR image; TDE, Target deviation error as the Euclidean distance between the planned and real (achieved) targets; TRE, Target registration error as Euclidean distance between the real-physical target point and its virtual counterpart (in mm); TVE3D, Target visualization error in 3D between the real-physical target point and its virtual counterpart (in mm).

Study	AR technology	Study typology	Method of accuracy evaluation	Reported accuracy
Maruyama et al. (32)	Epson Moverio (BT-200)	Phantom study	TRE over four target points at the border of a tumor	The mean and standard deviation were 2.1 and 1.1 mm respectively
Incekara et al. (33)	Microsoft HoloLens^®^	Patient study	TDE measured using a BrainLab neuronavigator as a gold standard	The overall median deviation between the two modalities was 4 mm with an interquartile range 0–0.8 mm
van Doormaal et al. (34)	Microsoft HoloLens^®^	Phantom and patient study	FRE calculated as the root-mean-square of the distance between skin fiducials	Phantom study: mean and standard deviation were 7.2 and 1.8 mm respectively. Patients: Mean and standard deviation were 4.4 and 2.5 mm respectively
McJunkin et al. (35)	Microsoft HoloLens^®^	Cadaver study and patient study	TRE over 7 pre-specified landmarks	Phantom study: the mean and standard deviation were 5.76 and 0.54 mm respectively
Li et al. (36)	Microsoft HoloLens^®^	Patient study	Postoperative CT scan used to measure the TDE in guiding the external ventricular drain	Mean and standard deviation of 4.34 and 1.63 mm
Fick et al. (37)	Microsoft HoloLens^®^	Patient study	FRE over 6 registrations	8.5 mm
Qi et al. (38)	Microsoft HoloLens^®^	Patient study	TDE measured using a BrainLab neuronavigator as a gold standard	The overall median deviation between the two modalities was 4.1 mm (IQR 3.0 mm–4.7 mm)

In this work, we compared the VOSTARS AR HMD to the StealthStation^®^ S7 EM navigation for accuracy in targeting superficial landmarks. Unlike most of the studies mentioned before, the performance of the AR HMD is not reported in terms of deviation from the traditional navigator measurements. The accuracy of both was evaluated by measuring the distance between the point targeted by the users and the real target with a caliper.

According to the study result, the VOSTARS platform yields a better performance both in vitro and in vivo.

The users involved in the phantom study performed significantly better with the VOSTARS system (median accuracy of 1.4 mm with VOSTARS and 2.9 mm with the StealthStation^®^ S7). Results obtained with VOSTARS align with our previous findings concerning estimating the real-to-virtual 3D target visualization error in a similar setup: the TVE3D mean and standard deviation were 1.3 and 0.6 mm, respectively.

Results obtained from in-vivo experiments showed a lower targeting accuracy with both guidance systems. This was to be expected since, for example, the patient’s anatomy at the time of testing may differ from the face model extracted from the MRI data set [see Type I error according to (9)]. In our specific protocol, this error may be higher than in traditional neuronavigation flow, as our study included healed patients who had MRIs up to six months before our experiments. Any changes that occurred between the time of the MRI data acquisition and the experiments could have reduced the accuracy of both navigation systems’ patient-to-image registration (as for the VOSTARS system, this could have determined modification to the fitting of the DRF on the patient’s facial anatomy).

Moreover, a limitation of the human study, is that only a a single user was recruited to perform the accuracy tests, thus reducing the number of tests performed to minimize patient discomfort. The test may also be biased due to its non-invasive nature. During the test, recruited volunteers were awake and their heads were not secured with a head clamp or holder. While subjects were instructed to remain as still as possible, any small movements that occurred during the test could have caused slight changes in the positioning of the reference frame of the StealthStation^®^/ the VOSTARS DRF on the subject face.

Additionally, both the targeting trial and the error measurements were more challenging in the in-vivo setup compared to the in-vitro setup. This is because both the user performing the navigation trial and the user measuring the errors needed to minimize contact with the volunteer’s face (with their hands and the pointer/caliper) to avoid discomfort.

In light of these factors, caution is advised when interpreting the absolute error values. The values obtained cannot be used to predict the accuracy of the VOSTARS system in a real surgical scenario. However, since all the errors mentioned affected both navigation systems in our protocol, the results obtained are useful for comparing the performance of the two systems.

The median targeting accuracy with the VOSTARS system was significantly better than that obtained with the StealthStation^®^ for selected landmarks at the level of the outer eyebrow (3.7 mm vs. 6.6, p = 0.05) and forehead (4.5 mm vs. 6.3, p = 0.021), while a non-significant difference was found for the pronasal point (2.7 mm vs. 3.5 mm, p = 0.123). Our findings suggest that the lower error in identifying this landmark might be attributed to its ease of anatomical localization. Conversely, the forehead landmark yielded the highest error, with the StealthStation^®^ tests reaching a maximum of 15.2 mm. This significant discrepancy is likely due, in part, to the inherent mobility of the skin on the forehead during targeting.

## 5 Conclusion and future scope

This study represents a critical preliminary step in validating the potential of the VOSTARS augmented reality (AR) system for neurosurgical navigation, towards its clinical use for guiding complex craniotomies. By comparing the VOSTARS platform to the established Medtronic StealthStation^®^ S7^®^ in both phantom and human studies, we have demonstrated the superior targeting accuracy of the AR system in superficial landmark localization. The phantom trials showed significantly higher accuracy with VOSTARS, confirmed in human volunteers.

Despite the positive results, this work is only an initial validation of the system’s capabilities. The in-vivo human study was limited in scope, with only one operator and no invasive surgical interventions. Furthermore, the tests were conducted under conditions more challenging than those outlined in the protocol planned for the future in vivo surgical trial. Specifically, the subject was awake and free to move, introducing significant variability and reducing accuracy compared to a controlled operative setting. Furthermore, the radiological images used for augmented reality data extraction could be up to six months old, potentially affecting registration accuracy due to anatomical or physiological changes over time.

In conclusion, despite the limitations of the study, the results obtained encourage further development of the VOSTARS platform. Thanks to this study’s results, in the coming months, we will expand this research with a comprehensive clinical trial designed to evaluate the in-vivo performance of VOSTARS during live craniotomies. This trial will assess the system’s accuracy in real-time surgical environments, focusing on complex craniotomies. We aim to explore its effectiveness in improving surgical precision, reducing operating time, and ultimately enhancing patient outcomes.

## Data Availability

The raw data supporting the conclusions of this article will be made available by the authors, without undue reservation.
